# Transcatheter management of obstructed baffle repairs of partial anomalous pulmonary veins: a case series

**DOI:** 10.1093/ehjcr/ytae203

**Published:** 2024-05-03

**Authors:** Thomas M Das, Patricia Blazevic, Nandini Mehra, Beka Bakhtadze, Joanna Ghobrial

**Affiliations:** Heart, Vascular, and Thoracic Institute, Cleveland Clinic Foundation, 9500 Euclid Ave, Office J2-142, Cleveland, OH 44195, USA; Heart, Vascular, and Thoracic Institute, Cleveland Clinic Foundation, 9500 Euclid Ave, Office J2-142, Cleveland, OH 44195, USA; Heart, Vascular, and Thoracic Institute, Cleveland Clinic Foundation, 9500 Euclid Ave, Office J2-142, Cleveland, OH 44195, USA; Heart, Vascular, and Thoracic Institute, Cleveland Clinic Foundation, 9500 Euclid Ave, Office J2-142, Cleveland, OH 44195, USA; Heart, Vascular, and Thoracic Institute, Cleveland Clinic Foundation, 9500 Euclid Ave, Office J2-142, Cleveland, OH 44195, USA

**Keywords:** Case series, Adult congenital heart disease, Partial anomalous pulmonary venous connections, Transcatheter therapies, Drug-coated balloons

## Abstract

**Background:**

Partial anomalous pulmonary venous return (PAPVR) can be surgically corrected using a pericardial baffle. This baffle can become obstructed post-surgery, leading to pulmonary hypertension and right heart dysfunction if not detected and corrected.

**Case summary:**

We describe three patients with occluded PAPVR baffles who underwent drug-coated balloon angioplasty and stenting of the obstructed baffle. In each case, baffle obstruction was detected post-operatively on surveillance cross-sectional imaging, and an invasively measured pulmonary capillary wedge-to-left atrium gradient was noted to be elevated. Post-intervention, each patient had an improvement in baffle flow by angiography as well as lung perfusion as assessed by nuclear medicine scintigraphy.

**Discussion:**

Given the subtle symptomatology of obstructed PAPVR pericardial baffle repairs, surveillance imaging is necessary to detect occluded baffles and intervene before downstream right heart disease and pulmonary hypertension develops. Given the high rates of re-stenosis in pulmonary vein stenting, pre-treatment of occluded PAPVR baffles with drug-coated balloons may help reduce re-intervention rates.

**Learning points**

- To appreciate the presentation and clinical sequelae of partial anomalous pulmonary venous return (PAPVR) pericardial baffle repair obstruction.
- To understand the utility of surveillance imaging after PAPVR pericardial baffle repair, given the risk of baffle obstruction and subtle symptomatology.
- To recognize the role of transcatheter interventions in obstructed pericardial baffle repairs for PAPVR.

## Introduction

Partial anomalous pulmonary venous return (PAPVR) describes a spectrum of congenital heart defects characterized by connections of one or more pulmonary veins to the right atrium (RA) or systemic veins with an overall incidence of 0.7% based on autopsy series.^1^ This aberrant pulmonary drainage can lead to right heart dilation and dysfunction, necessitating surgical repair to re-route pulmonary venous blood into the left atrium (LA).^2^ Many surgical techniques have been proposed to repair the various PAPVR anatomies, including the use of a pericardial patch to baffle blood through the inter-atrial septum, and the *in situ* ‘pericardial roll’ repair for PAPVR connections distal to the LA such as the superior or middle aspects of the superior vena cava (SVC).^3,4^ One well-described complication of these surgeries is obstruction of the pericardial baffle, necessitating re-intervention.^5,6^ Post-surgical baffle obstruction can present late due to subtle symptomology. When left untreated, it can lead to pulmonary hypertension and right heart failure due to pulmonary vein intimal thickening and fibrosis, with upstream vascular remodelling extending into the pulmonary arterial tree. Such intimal thickening has been demonstrated in pericardial baffles used in the surgical repair of veins and arteries.^7,8^

One therapeutic option to avoid repeat open heart surgery is percutaneous stenting.^9^ We describe our experience with three adults who underwent stent implantation in PAPVR baffle obstruction, with the novel use of drug-coated balloon angioplasty to deliver anti-proliferative drug therapy in the pericardial baffle.

## Summary figure

Case summaries and representative CT imaging of three patients with occluded partial anomalous pulmonary venous return (PAVPR) pericardial baffle repairs who underwent transcatheter drug-coated balloon (DCB) angioplasty and stenting. Pre-intervention CT imaging (left) shows occluded baffle repairs (red arrows), and post-intervention CT imaging (right) shows patency of the stented baffles (left).

**Figure:**
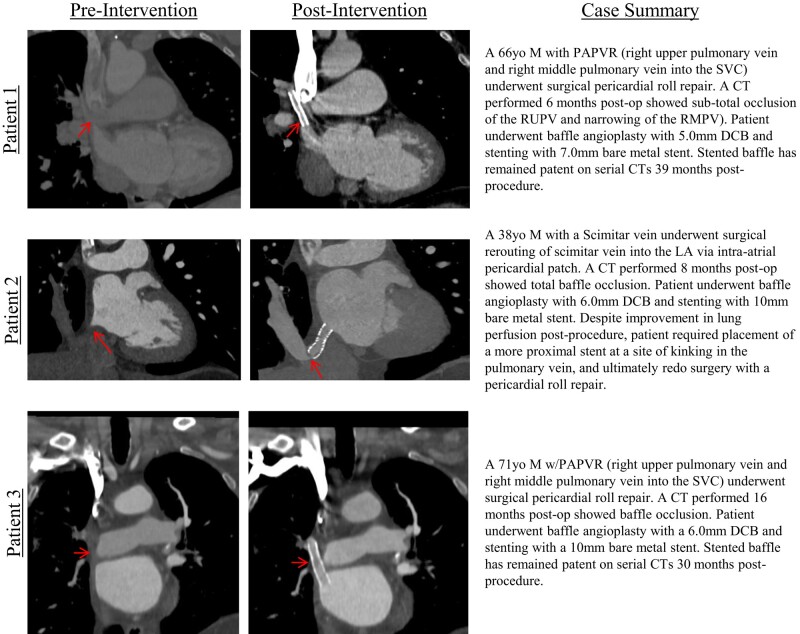


### Patient 1

**Figure 1:**
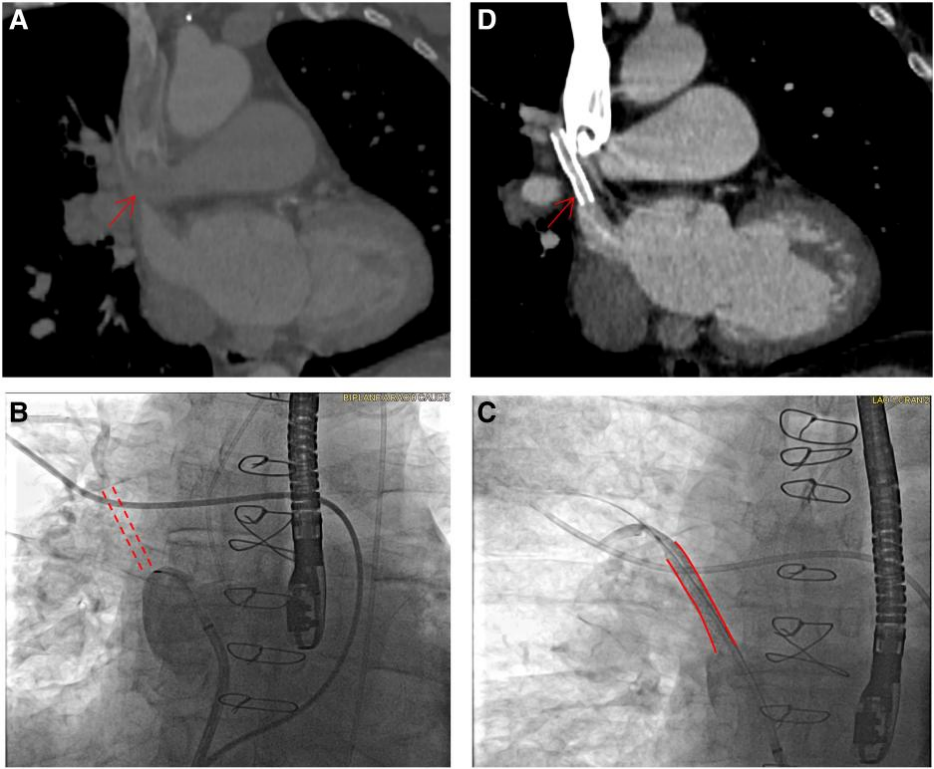
Case 1. (*A*) Computed tomography scan 6 months post-surgery showing obstructed baffle (arrow). (*B*) Fluoroscopy showing obstructed baffle (dotted lines). (*C*) Fluoroscopy showing patent baffle post-stenting (solid lines). (*D*) CT at 39 months post-stenting showing patent baffle.

A 66-year-old man with a history of paroxysmal atrial fibrillation, hypertension, and sleep apnoea presented with worsening dyspnoea on exertion and fatigue. Transthoracic echocardiogram showed mildly depressed right ventricular (RV) function qualitatively as well as RA and RV enlargement. Cardiac magnetic resonance imaging (MRI) revealed PAPVR with drainage of the right upper pulmonary vein (RUPV) and right middle pulmonary vein (RMPV) into the SVC, severe dilation of the RV [RV end-diastolic volume index (RVEDVi) 155 mL/m^2^ and normal 60–106 mL/m^2^] with normal RV function, and a *Qp*:*Qs* of 2.0. He underwent a surgical pericardial roll repair with concurrent maze procedure and excision of the atrial appendages. After surgery, he was adherent to a regimen of aspirin 81 mg daily and apixaban 5 mg twice daily. A surveillance cardiac computed tomography scan (CT) performed 6 months post-op showed subtotal occlusion of the baffle and RUPV, as well as narrowing of the RMPV (*Figure 1A*). The patient, previously able to exercise vigorously, now reported new limitations since surgery. In clinic, he was hypertensive with a blood pressure of 151/74 mmHg, a heart rate of 75 b.p.m., and an oxygen saturation of 97%. Physical exam revealed no evidence cyanosis or volume overload, and no murmurs or RV heave was appreciated. He was referred for percutaneous intervention.

The procedure was performed under general anaesthesia and with transesophageal echocardiogram (TEE) guidance. Access included a 7F sheath in the right common femoral vein for right heart catheterization, a 6F sheath in the right common femoral artery for arterial monitoring, and a 9F sheath in the left common femoral vein for the intervention. Transeptal puncture was performed to gain access to the LA. Notably, all cases in this report had a similar approach to vascular access. The occluded pericardial roll baffle was engaged (*Figure 1B*; Supplementary material online, *Video S1A*). The invasively measured gradient from the mean right upper pulmonary artery wedge pressure to the mean LA pressure was 17 mmHg. The lesion was wired in a retrograde fashion and dilated with a 4.0 × 40 mm balloon and then a 5.0 × 40 mm drug-coated balloon (DCB) (IN.PACT Admiral, Medtronic) and then stented with a 7 × 39 mm bare metal stent (Omnilink, Abbott). TEE confirmed normalized pulmonary vein and baffle flow. Right upper lung to LA gradient improved from 17 to 0 mmHg, and angiography revealed unobstructed baffle flow (*Figure 1C*; Supplementary material online, *Video S1B*).

Serial post-procedure CT scans have demonstrated stent patency up to 39 months (*Figure 1D*), with resolution of symptoms. Pre- and post-procedure lung perfusion scintigraphy studies using IV technetium 99 showed relatively improved right lung perfusion from 51% to 56%.

### Patient 2

**Figure 2:**
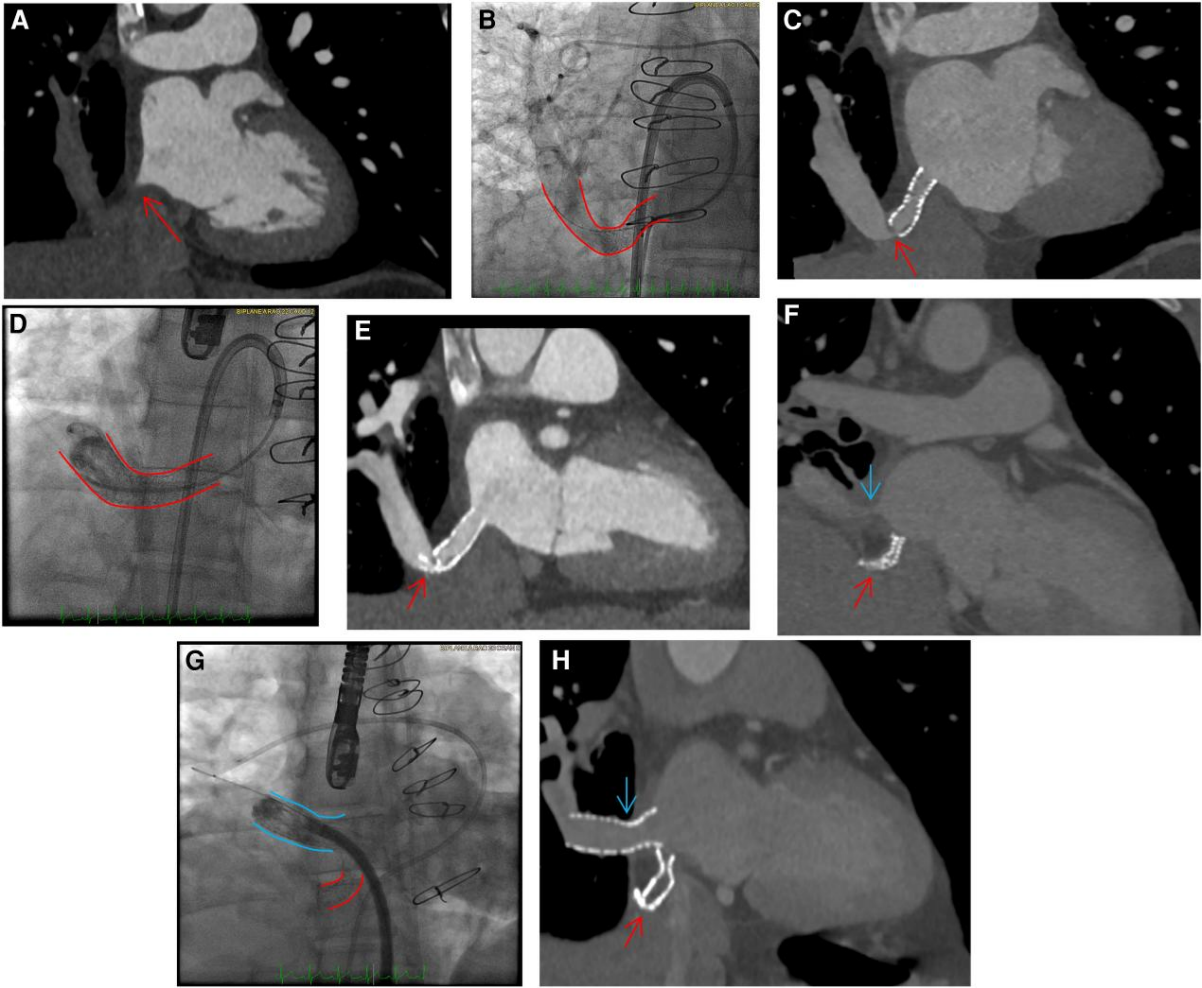
Case 2. (*A*) Computed tomography scan at 8 months post-surgery showing obstructed baffle (arrow). (*B*) Fluoroscopy showing patent baffle following intervention (solid line indicating baffle). (*C*) Computed tomography scan at 12 months post-stenting showing kinking of the vein (arrow). (*D*) Fluoroscopy from second intervention showing patent baffle after second stent (solid lines indicating baffle). € Computed tomography scan at 11 months after second stenting procedure showing crushed stent (arrow). (*F*) Computed tomography scan at 6 months after re-do surgery with pericardial roll showing obstructed original baffle (lower arrow) and obstructed new pericardial roll (upper arrow). (*G*) Fluoroscopy showing first obstructed baffle (lower solid lines) and now patent pericardial roll post-stenting (upper solid lines). (*H*) Computed tomography scan at 6 months after intervention showing patent-stented pericardial roll baffle (upper arrow) and obstructed original baffle (lower arrow).

A 38-year-old man with a scimitar vein (drainage of right-sided pulmonary veins into the right atrium/inferior vena cava junction) presented with fatigue and decreased exercise tolerance. Studies performed at the referring facility demonstrated an invasively measured *Qp*:*Qs* of 3:1 and RA and RV enlargement. He underwent surgical re-routing of the scimitar vein into the LA via intra-atrial pericardial patch through the fossa ovalis. After surgery, he was adherent to a medical regimen of aspirin 81 mg daily. While he reported initial symptom improvement, at 8-month follow-up, he reported worsening of his exercise tolerance. In clinic, he was hypertensive with a blood pressure of 143/75 mmHg, heart rate of 82 b.p.m., and oxygen saturation of 96%. The patient was euvolemic on physical exam, and no cyanosis, murmurs, or RV heave was appreciated. Computed tomography scan demonstrated total occlusion of the baffle repair (*Figure 2A*). He was referred for percutaneous intervention.

In the catheterization lab, right pulmonary artery angiography revealed a lack of right lung venous drainage, consistent with baffle occlusion (see Supplementary material online, *Video S2A*). The gradient from the mean right upper pulmonary artery wedge pressure to the mean LA pressure was 12 mmHg. The scimitar vein baffle was serially dilated with 3 × 20 mm and 4 × 20 mm semi-compliant balloons before subsequent dilation with a 6 × 40 mm DCB and then stented with a 10 × 29 mm Omnilink bare metal stent. Angiography revealed patency of the scimitar baffle stent (*Figure 2B*; Supplementary material online, *Video S2B*).

Post-procedure CT showed a patent baffle stent with good contrast opacification; however, a kink in the pulmonary vein itself distal to the stent was noted as it takes an acute angulation. Lung perfusion scintigraphy showed improved right lung perfusion from 8% to 21%.

Repeat CT performed 12 months post-procedure due to worsening dyspnoea on exertion showed reduced flow at the site of the acute baffle angulation (*Figure 2C*). He subsequently underwent repeat catheterization; angiography revealed obstruction of baffle flow into the LA at that angulated site (see Supplementary material online, *Video S2C*). Stent extension with an additional overlapping 10 × 19 mm bare metal stent deployed distal to the first stent at the site of angulation was performed to maintain baffle patency (*Figure 2D*; Supplementary material online, *Video S2D*). While initially successful, repeat CT 11 months after the second procedure showed stent compression at the site of acute angulation (*Figure 2E*). Given symptoms and concern for compromised pulmonary drainage of the entire right lung, he then underwent re-do surgery with a pericardial roll baffle repair. Surveillance CT 6 months after his second surgery showed the pericardial roll baffle was subtotally occluded at its anastomosis to the LA and repeat right pulmonary artery angiography confirmed a lack of contrast drainage (*Figure 2F*; Supplementary material online, *Video S2E*). He ultimately underwent percutaneous angioplasty with 6.0 × 40 mm and 7.0 × 40 mm DCBs and stenting with a 36 × 12 mm Ev3 mounted on a 14 mm balloon-in-balloon system, with restoration of flow by angiography (*Figure 2G*; Supplementary material online, *Video S2F*). This stent has remained patent on CT at 6-month follow-up (*Figure 2H*).

### Patient 3

**Figure 3:**
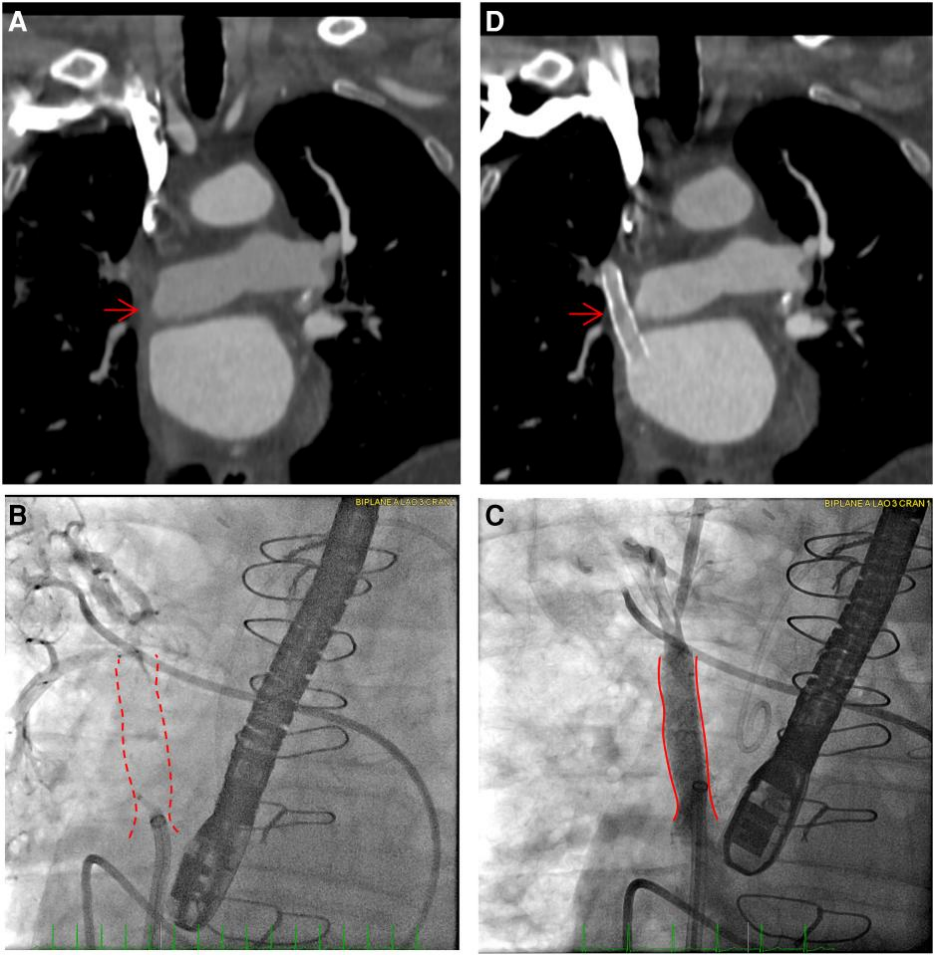
Case 3. (*A*) Computed tomography scan at 16 months post-surgery with obstructed baffle (red arrow). (*B*) Fluoroscopy showing obstructed baffle (red dotted lines). (*C*) Post-procedure fluoroscopy showing patent baffle (red solid lines). (*D*) Computed tomography scan performed 30 months post-stenting showing patent baffle (red arrow).

A 71-year-old man with a history of atrial fibrillation, hypertension, and PAPVR involving drainage of the RUPV and RMPV into the mid-SVC presented with dyspnoea. Transthoracic echocardiogram showed normal biventricular function with RA and RV dilation. Cardiac magnetic resonance imaging subsequently showed evidence of RV enlargement (RVEDVi 128 mL/m^2^) and a *Qp*:*Qs* of 1.8:1. He underwent uncomplicated pericardial roll repair of the anomalous pulmonary veins and patch repair of the SVC. After surgery, he was adherent to a regimen of apixaban 5 mg twice daily. Surveillance CT performed 16 months post-op showed baffle occlusion (*Figure 3A*). He denied subjective symptoms at the time. In clinic, he was normotensive with a blood pressure of 110/56 mmHg, heart rate of 65 b.p.m., and oxygen saturation of 98%. Physical exam showed no evidence of cyanosis, volume overload, or cardiac murmurs. Given concern regarding the risk of developing pulmonary hypertension in the setting of obstructed pulmonary vein flow, he was referred for percutaneous intervention.

In the catheterization lab, the occluded baffle was engaged and wired (*Figure 3B*; Supplementary material online, *Video S3A*). The invasively measured gradient from the mean right upper pulmonary artery wedge pressure to the mean LA pressure was 13 mmHg. The occluded baffle was dilated with a 4.0 × 2.0 mm semi-compliant balloon, followed by a 6.0 × 40 mm DCB. The baffle was then stented with a 10 × 39 mm Omnilink bare metal stent with angiography confirming unobstructed flow (*Figure 3C*; Supplementary material online, *Video S3B*). The baffle stent remains patent on CT at 30 months post-procedure (*Figure 3D*). Lung perfusion scintigraphy showed improved right lung perfusion from 41% to 49%.

## Discussion

We present three patients who underwent DCB angioplasty and stenting of a PAPVR baffle occlusion (*Table 1*). Each patient initially presented with PAPVR and right heart enlargement and underwent surgical pericardial baffle creation, re-routing the anomalous pulmonary veins into the LA. Follow-up CT (performed over a mean of 10 months post-operatively) confirmed baffle obstruction. As described in the 2020 European Society of Cardiology (ESC) guidelines for the management of adult congenital heart disease, the risk of post-operative thrombosis and occlusion of an operated upon anomalous pulmonary vein remain small but persistent.^10^ There is no consensus on medical therapy after surgical PAPVR repair, and each patient was on a different anti-thrombotic regimen post-operatively. When left undetected, PAPVR pericardial baffle occlusion can lead to significant morbidity by upstream vascular remodelling, with subsequent risk of pulmonary hypertension, right heart failure, and limiting symptoms. It can be difficult to detect baffle obstruction due to its subtle symptomatology. Therefore, routine post-operative imaging is imperative to avoid irreversible damage and long-term clinical sequelae. Either cardiac CT or MRI could be considered for this surveillance, with the caveat that CT may be more accurate in assessing for occlusion of small calibre baffles at the cost of additional radiation. The appropriate intervals for surveillance imaging to detect occult baffle obstruction are an area of ongoing investigation.

**Table 1:** Clinical, procedural, and outcome data

	Brief history	Veins involved	Pre-intervention NM lung perfusion results	Pre-intervention haemodynamics	Intervention	Post-intervention NM lung perfusion results	Outcome
**Patient 1**	A 66-year-old M with PAPVR (RUPV and RMPV into the SVC) underwent surgical pericardial roll repair. A CT performed 6 months post-op showed subtotal occlusion of the RUPV and narrowing of the RMPV. Patient reported decreased tolerance.	Right upper and right middle	Total perfusion to right lung—51%Total perfusion to left lung—49%	Mean PA—35 mmHgMean LPA wedge—20 mmHg Mean RPA wedge—35 mmHg Mean LA—18 mmHg	Baffle angioplasty with 5.0 drug-coated balloon and stenting with a 7.0 × 39 mm Omnilink stent	Total perfusion to right lung—56%Total perfusion to left lung—44%	Stent baffle has remained patent to date (39 months post-procedure)
**Patient 2**	A 38-year-old M with a scimitar vein underwent surgical re-routing of scimitar vein into the LA via intra-atrial pericardial patch. A CT performed 8 months post-op showed total baffle occlusion. Patient reported decreased exercise tolerance.	All right-sided pulmonary veins	Total perfusion to right lung—8%Total perfusion to left lung—92%	Mean PA—26 mmHgMean LPA wedge—14 mmHgMean RPA wedge—26 mmHgMean LA—14 mmHg	Baffle angioplasty with 6.0 drug-coated balloon and stenting with a 10.0 × 29 mm Omnilink stent	Total perfusion to right lung—21%Total perfusion to left lung—79%	Required additional stent due to kinking in pulmonary vein, followed by surgical revision with pericardial roll repair, followed by stenting in pericardial roll baffle, which is patent at 6-month follow-up.
**Patient 3**	A 71-year-old M w/PAPVR (RUPV and RMPV into the SVC) underwent surgical pericardial roll repair. A CT performed 16 months post-op showed baffle occlusion. Patient denied symptoms despite baffle obstruction.	Right upper and right middle	Total perfusion to right lung—41%Total perfusion to left lung—59%	Mean right lower PA wedge—17 mmHgMean right upper PA wedge—30 mmHgMean LA—17 mmHg	Baffle angioplasty with 6.0 drug-coated balloon and stenting with a 10.0 × 39 mm Omnilink stent	Total perfusion to right lung—49%Total perfusion to left lung—51%	Stent baffle has remained patent to date (30 months post-procedure)

The longer a PAPVR pericardial baffle is obstructed, the greater the technical challenge in recanalization. Moreover, tissue remodelling over time limits lumen diameter and stent size optimization. Since the rate of in-stent restenosis of pulmonary veins remains high, a combination of larger stents (currently only available in the bare metal form) and drug delivery using DCB may prove to be a suitable combination to improve the rate of long-term patency and reduce the re-intervention rate.^11^ While the use of DCB angioplasty has been reported in pulmonary vein stenosis following radiofrequency ablation, the use of DCB in anomalous pulmonary veins and pericardial baffle repairs has not been reported in the literature.^12^

Over a median follow-up of 12 months, one patient (Patient 2) required a repeat intervention due to the acute angulation of the baffle leading to stent compression, not in-stent restenosis, and did require repeat surgery and, ultimately, repeat stenting. The other two patients have been free from repeat intervention to date. In each case, lung perfusion scintigraphy showed an objective improvement in lung perfusion following intervention.

## Conclusion

Transcatheter management is a feasible option for patients with occluded PAPVR pericardial baffle repairs, a known complication post-surgical repair, obviating the need for re-do sternotomies. The addition of DCB angioplasty is a promising therapeutic modality that may reduce the re-intervention rate due to in-stent restenosis. These cases underscore the importance of routine imaging for early detection of baffle obstruction to reduce the risk of future morbidity. Drug-coated balloon angioplasty and stenting contribute to the available armamentarium in the management of PAPVR surgical repair complications. Long-term follow-up is warranted to ascertain its durability and impact on long-term morbidity. This is the first reported case series description of successful DCB angioplasty and stenting of occluded pericardial baffles post-surgical PAPVR repair, with encouraging short-term outcomes.

## Supplementary Material

ytae203_Supplementary_Data

## Data Availability

The data underlying this article are available in the article and in its online supplementary material.

## Lead author biography

Dr Thomas M. Das is a cardiology fellow at the Cleveland Clinic in Cleveland, OH. His clinical interests include adult congenital heart disease and interventional cardiology. He is also passionate about cardiovascular medical education and serves as program director for the CardioNerds Academy medical education program (https://nam12.safelinks.protection.outlook.com/?url=3Dhttps%3A%2F%2Fwww.cardionerds.com%2Facademy%2F&data=3D05%7C02%7Coup2nts%40novatechset.com%7Cd55c57e2e13d4cee17ee08dc5e348067%7Ca03a7f6cfbc84b5fb16bf634dbe1a862%7C0%7C0%7C638488826811412768%7CUnknown%7CTWFpbGZsb3d8eyJWIjoiMC4wLjAwMDAiLCJQIjoiV2luMzIiLCJBTiI6Ik1haWwiLCJXVCI6Mn0%3D%7C0%7C%7C%7C&sdata=3Dh4MwLXU3mtgt74CsIQwteEcP3vAJzv8wr03cRKMwXXM%3D&reserved=3D0).

## Supplementary material

**Consent:** The authors confirm that written consent for the submission and publication of this case report including the images and associated text have been obtained from the patient in line with COPE guidance.

**Funding:** No external funding contributed to this work
